# Effect of Uncomplicated Cataract Surgery on Central Macular Thickness in Diabetic and Non-diabetic Subjects

**DOI:** 10.18502/jovr.v14i4.5447

**Published:** 2019-10-24

**Authors:** Brahm Prakash Guliani, Isha Agarwal, Mayuresh P. Naik

**Affiliations:** ^1^Department of Ophthalmology, Vardhaman Mahavir Medical College & Safdarjung Hospital, New Delhi, India; ^2^Department of Ophthalmology, Hamdard Institute of Medical Sciences & Research, Hakeem Abdul Hameed Centenary Hospital, New Delhi, India

**Keywords:** Central Macular Thickness, Diabetic Macular Edema, Uncomplicated Phacoemulsification

## Abstract

**Purpose:**

To assess the quantitative changes of macula in diabetic and non-diabetic eyes after uncomplicated cataract surgery.

**Methods:**

In this prospective interventional study being performed in a tertiary healthcare hospital, a total of 660 eyes were divided into two groups. Group 1 included 330 eyes from healthy subjects and group 2 included 330 eyes from well-controlled diabetic subjects with no diabetic retinopathy planned for phacoemulsification with foldable IOL implantation by the same surgeon under similar settings. Optical Coherence Tomography (Heidelberg Spectralis SD-OCT) was used to assess preoperative and postoperative central macular thickness (CMT) at weeks 1 and 6.

**Results:**

The mean CMT in group 1 preoperatively, at postoperative week 1, and at post-operative week 6 was 257.03 ± 20.904, 262.82 ± 17.010, and 265.15 ± 20.078 µm, respectively. The corresponding values in group 2 were 255.36 ± 17.852, 259.15 ± 16.644, and 266.09 ± 18.844 µm, respectively. There was no significant difference in the mean CMT values between the two groups on any of the three occasions when the CMT was measured (*P* = 0.374 and *P* = 0.313 at weeks 1 and 6, respectively).

**Conclusion:**

There was no statistically significant difference in CMT between normal subjects and diabetic subjects without diabetic retinopathy preoperatively and in early postoperative period after uncomplicated phacoemulsification surgery.

##  INTRODUCTION 

Cataract extraction is one of the most commonly performed ophthalmic surgeries. Recent innovations in instrumentation, lens design, and surgical technique have improved the outcome of cataract surgery.^[1]^ Currently, the preferred technique is phacoemulsification using small incisions and implantation of a foldable intraocular lens (IOL).^[2]^ This is an efficient procedure, and uneventful surgery is generally associated with good visual results.^[3]^ However, the development of cystoid macular edema (CME) can lead to suboptimal postoperative vision.^[4]^ This can occur in patients with ocular diseases, such as uveitis or diabetic retinopathy (DR), even after uncomplicated cataract surgery.^[5]^

CME following cataract surgery was initially reported by Irvine in 1953 and demonstrated angiographically by Gass and Norton in 1966 and has come to be known as the Irvine Gass syndrome.^[6]^

It is not uncommon to encounter CME in otherwise healthy eyes after uneventful phacoemulsification surgery.^[7]^ The incidence after phacoemulsification is reported to be 0.1–2% in healthy populations.^[7]^

Although the exact pathophysiology is not known, the role of surgical trauma with the release of prostaglandins and blood–retinal barrier disruption is suspected.^[8]^ Light toxicity and vitreomacular traction might also have a role.^[9]^

The incidence of pseudophakic CME depends on the methodology used in its detection. It has been suggested that prophylactic use of non-steroidal anti-inflammatory drugs preoperatively, and steroids and anti-inflammatory drugs in the postoperative period, reduces the incidence of postoperative pseudophakic CME.^[10]^

Diabetes mellitus increases the probability of developing cataract and the risk of decreased visual outcomes after cataract surgery.^[7]^

It has been suggested that in diabetics macular edema after cataract surgery occurs predominantly in those with concurrent pre-existing diabetic macular edema (DME) involving the center of the macula.^[11]^ While others have reported that the development of postoperative macular edema does not need pre-existing DME,^[11]^ these were published before the availability of optical coherence tomography (OCT) technology. The dynamics of macular edema and cataract surgery in those with DR can be explored using the qualitative and quantitative OCT-based data.

Here we evaluate the retinal thickness changes in the early postoperative course of six weeks in non-diabetic and diabetic subjects after uncomplicated phacoemulsification with intracapsular IOL implantation.

##  METHODS

Ethical clearance was obtained from the Ethics Committee, the Institutional Review Board at Vardhaman Mahavir Medical College (V.M.M.C), and the Safdarjung Hospital, New Delhi.

This tertiary health center-based observational study was conducted on adult patients posted for cataract surgery at the Department of Ophthalmology, VMMC and Safdarjung Hospital, New Delhi. Assuming the effect size to be 0.8 (ratio of difference of two means/standard error), power to be 85%, and the level of significance to be 5%, a sample size of 330 eyes per group, including the 10% loss to follow-up, was required for the study, using software G power 3.1.

The patients were divided into two groups based on the inclusion and exclusion criteria as follows: GROUP 1, 330 healthy subjects planned for phacoemulsification with foldable IOL implantation by the same surgeon under similar settings; GROUP 2, 330 well-controlled diabetic subjects with no DR posted for phacoemulsification with foldable IOL implantation by the same surgeon under similar settings.

The inclusion criteria for group 1 (healthy subjects) included age > 40 years and senile cataract undergoing uncomplicated cataract surgery. Patients with complicated cataract surgery, intraocular pressure > 21 mmHg, dense white cataract in whom OCT could not be performed, and any ocular diseases that might influence central macular thickness (CMT), such as glaucoma, uveitis, and age-related macular degeneration, were excluded from the study. Patients with a history of previous eye surgery or a history of macular edema in the fellow eye were also excluded.

Group 2 (well-controlled diabetic subjects) had similar inclusion criteria along with a diagnosis of diabetes mellitus of any duration, controlled on oral hypoglycemic agents or insulin, blood sugar < 200 mg% (All-India-Ophthalmological-Society (AIOS) Guidelines to Prevent Intraocular Infection, 2009) and HbA1C < 7% (American Diabetes Association ADA A1C Goals, Standards of Medical Care in Diabetes-2015), and an absence of any evidence of DR as assessed by indirect ophthalmoscopy and OCT.^[12]^ Besides similar exclusion criteria as for group 1, group 2 patients were also excluded based on the presence of anemia [men (> 15 yr), < 13 g/dL; women (> 15 yr), < 12 g/dL],^[13]^ pregnancy, or diabetic nephropathy (abnormal kidney function test including serum electrolytes, microalbuminuria, serum creatinine, and serum urea).

OCT (Heidelberg Spectralis SD-OCT) was used to assess pre- and postoperative (at weeks 1 and 6) CMT (central subfield thickness equating to mean thickness in the central 1000-μm diameter area).

Repeated measure analysis and multiple comparison correction with the Bonferroni method were applied to the data. *P* < 0.05 was considered statistically significant.

##  RESULTS

The majority of the patients (47%) were in the age group of 51–60 years [Table 1]. More than half of the patients in group 1 (54.5%) and 39.4% of patients in group 2 were between 51 and 60 years old (no significant difference when comparing groups, *P* = 1.71).

**Table 1:** Age- and sex-wise distribution of study subjects among the two study groups


**Age Groups (Years)**	**GROUP 1**		**GROUP 2**	
	**Male**	**Female**	**Male**	**Female**
41–50	30 (9.09%)	20 (6.06%)	20 (6.06%)	10 (3.03%)
51–60	100 (30.30%)	80 (24.24%)	70 (21.21%)	60 (18.18%)
61–70	40 (12.12%)	40 (12.12%)	50 (15.15%)	40 (12.12%)
> 70	15 (4.54%)	5 (1.51%)	40 (12.12%)	40 (12.12%)
Total	170 (51.5%)	160 (48.5%)	220 (66.7%)	110 (33.3%)
Total	330 (100%)		330 (100%)	
	
	

The mean age in group 1 was 58.30 ± 7.66 years, while the mean age in group 2 was 63.24 ± 9.74 years (*P* = 2.89).

Overall, the study comprised of 390 (59%) males and 270 (41%) females [Table 1]. Group 1 comprised of 51.5% males and 48.5% females and group 2 of 66.7% males and 33.3% females (*P* = 2.12).

The mean CMT values in group 1 preoperatively and at postoperative weeks 1 and 6 were 257.03 ± 20.90, 262.82 ± 17.01, and 265.15 ± 20.07 µm, respectively [Table 2]. The mean CMT changes in group 1 preoperatively versus postoperative week 1 versus postoperative week 6 were statistically significant (both *P*-values < 0.001). The mean CMT changes at postoperative weeks 1 and 6 were also significantly different (*P*-value < 0.001).

**Table 2:** Groups 1 and 2: Repeated measure analysis and multiple comparison correction with Bonferroni method depicting change in mean central macular thickness from baseline to postoperative week 1 and week 6


	**GROUP 1**	**GROUP 2**	
Pre-op CMT	257.03 ± 20.904	255.36 ± 17.852	
Week 1 post-op CMT	262.82 ± 17.010	259.15 ± 16.644	
Week 6 post-op CMT	265.15 ± 20.078	266.09 ± 18.844	
Change in mean CMT at week 1 post-op as compared to pre-op baseline	5.788 ± 11.324 (*P*-value 0.006 for INTRAGROUP COMPARISON)	3.788 ± 6.066 (*P*-value 0.001 for INTRAGROUP COMPARISON)	*P*-value 0.374 for INTERGROUP COMPARISON
Change in mean CMT at week 6 post-op as compared to pre-op baseline	8.121 ± 11.056 (*P*-value < 0.001 for INTRAGROUP COMPARISON)	10.727 ± 9.722 (*P*-value < 0.001 for INTRAGROUP COMPARISON)	*P*-value 0.313 for INTERGROUP COMPARISON
Change in mean CMT at week 6 post-op as compared to week 1 post-op	2.333 ± 9.504 (*P*-value 0.172 for INTRAGROUP COMPARISON)	6.939 ± 7.208 (*P*-value < 0.001 for INTRAGROUP COMPARISON)	*P*-value 0.336 for INTERGROUP COMPARISON
	
	
CMT, central macular thickness, pre-op, preoperative; post-op, postoperative			

The mean CMT values in group 2 preoperatively, at postoperative week 1, and at postoperative week 6 were 255.36 ± 17.85, 259.15 ± 16.64, and 266.09 ± 18.84 µm, respectively [Table 2].

The mean CMT changes in group 2 preoperatively versus postoperative week 1 versus postoperative week 6 were both statistically significant (*P*-value < 0.001). The mean CMT changes at postoperative weeks 1 and 6 were also significantly different (*P*-value < 0.001).

No significant difference was noted in the mean CMT values between the two groups on any of the three occasions when CMT was measured [Table 2]. There was no significant change in the variation of mean CMT at weeks 1 and 6 postoperatively from baseline when groups 1 and 2 were compared [Table 2].

In our study, none of the patients developed clinical macular edema or CME on OCT.

##  DISCUSSION

This prospective comparative study was undertaken to assess the effect of uncomplicated phacoemulsification procedure with IOL implantation on CMT in diabetic and nondiabetic subjects in the early postoperative period (up to six weeks). The CMT used for comparison among the study subjects in our study corresponded to the mean thickness of all points in the central subfield of 1-mm diameter of the ETDRS macular subfields. The CMT was assessed with OCT preoperatively, and at weeks 1 and 6 postoperatively, and comparisons were made between the measurements of the two study groups. The macula in healthy controls as well as in controlled diabetics without DR was increased significantly at the end of the first and sixth weeks postoperatively compared to the preoperative results. In both groups, this thickening persisted until six weeks postoperatively in all subjects and did not regress to preoperative levels till the last follow-up at six weeks. This study demonstrated that the influence of uncomplicated cataract surgery on CMT in well-controlled diabetic patients without DR did not significantly differ from healthy non-diabetic subjects after uncomplicated cataract surgery. In other words, well-controlled diabetics without DR and nondiabetic patients showed similar intragroup thickening of the central macular subfield at weeks 1 and 6 after uncomplicated phacoemulsification, and the intergroup comparison was not statistically significant.

The rate of development of macular edema following cataract surgery at different time intervals in people with diabetes (with or without DR) varies from 31% to 81%.^[14]^ Certain minimal changes in the retina like subclinical CME and retinal leakage can occur even after uneventful cataract surgery. These subclinical changes in macular thickness after cataract surgery can be easily diagnosed on OCT and fluorescein angiography (FA).^[15]^ It has been reported by some studies that macular edema after cataract surgery, in people with diabetes, may occur predominantly in patients with concurrent pre-existing DME involving the center of the macula. On the other hand, some researchers have reported that for postoperative macular edema to develop, pre-existing DME is not required.^[11]^ However, these studies were completed prior to the availability of OCT technology. For detecting CME, the sensitivity and specificity of OCT is 96% and 100%, respectively, compared with FA.^[16]^ OCT can detect not only macular thickening before any angiographic evidence of macular edema but also produces reproducible and consistent quantitative results that are ideal for follow-up and assessment of the treatment response.^[16,17]^ For these reasons, we chose OCT as the investigative modality in our observational study.

There is some disagreement in the observations of various studies reporting an increase in CMT or development of macular edema after cataract surgery in patients with diabetes without DR. In a case-control study conducted on around 4,500 diabetics without preoperative macular edema, the incidence of postoperative macular edema was 4%, which was higher than that in the population without diabetes (*P* < 0.001).^[17]^ These authors also reported a higher risk for the development of macular edema (RR 1.80) in diabetic subjects without DR compared to patients without diabetes (RR 1.17).^[17]^ On the other hand, Katsimpris et al found increased macular thickness after uncomplicated cataract surgery in diabetics without DR compared to preoperative values or to a control group of patients at all follow-ups up to 12 months after cataract surgery.^[18]^ The eyes of diabetic patients without DR presented higher CMT and a higher incidence of CME after cataract surgery compared to the eyes of healthy controls, thus explaining the unsatisfactory visual acuity following cataract surgery in these patients.^[18]^ However, a recently conducted meta-analysis among diabetic patients without DR observed no statistically significant increase in CMT values after cataract surgery at one, three, and six months after cataract extraction.^[19]^

Many studies have postulated an association between progression of DR and cataract surgery,^[20]^ whereas other studies did not observe any significant association and consider any diabetic retinal changes as part of the natural course of the disease.^[21]^

In our study, the preoperative CMT measured by OCT is the same between the two groups. This is in accordance with a study conducted by Massin et al, who also found no differences in macular thickness comparing healthy subjects and diabetics without CME.^[22]^

There are, unfortunately, three limitations in our study. First, there are certain variables affecting the quality of OCT, despite it being a fast, non-invasive, non-contact, reproducible, and reliable in-vivo imaging technique.^[23]^ When media opacities, such as cataract, are present (especially in the form of cortical and subcapsular types), reliable scans might not be obtained preoperatively.^[23]^ To avoid this difficulty, we excluded patients with dense media opacities. Second, though our study showed no significant statistical difference between the two groups, few other studies have shown a significant rise in the CMT postoperatively in well-controlled diabetics with no DR. Therefore, large-scale studies with a longer follow-up period are likely required to accurately elucidate the role of diabetes control and DR status on the postoperative visual prognosis of patients undergoing uncomplicated phacoemulsification. Last, the current study is limited by the duration of follow-up of patients that precludes any firm clinical conclusions based on the results of the study.

In conclusion, CMT is increased after uncomplicated phacoemulsification both at weeks 1 and 6 postoperatively in both healthy nondiabetic subjects and in well-controlled diabetic patients without DR; the difference between the two groups is not statistically significant. It is postulated that good diabetes control is needed to prevent an increase in CMT and postoperative macular edema after uncomplicated uneventful phacoemulsification procedure. However, long term follow-up studies may be required so that management algorithms can be formulated in order to dictate our surgical paradigms.

##  Financial Support and Sponsorship

Nil.

##  Conflicts of Interest

There are no conflicts of interest.

## References

[1] DeCroos F. C., Afshari N. A. (2008). Perioperative antibiotics and anti-inflammatory agents in cataract surgery. *Current Opinion in Ophthalmology*.

[2] Panchapakesan J., Rochtchina E., Mitchell P. (2004). Five-year change in visual acuity following cataract surgery in an older community: the Blue Mountains Eye Study. *Eye*.

[3] Linebarger E. J., Hardten D. R., Shah G. K., Lindstrom R. L. (1999). Phacoemulsification and modern cataract surgery. *Survey of Ophthalmology*.

[4] (2005). Correction to: O’Brien, TP. Emerging guidelines for use of NSAID therapy to optimize cataract surgery patient care. Curr Med Res Opin 2005; 21(7):1131–37. doi:10.1185/030079905X50651. *Current Medical Research and Opinion*.

[5] Nelson M. L., Martidis A. (2003). Managing cystoid macular edema after cataract surgery. *Current Opinion in Ophthalmology*.

[6] Nelson M. L., Martidis A. (2003). Managing cystoid macular edema after cataract surgery. *Current Opinion in Ophthalmology*.

[7] GASS J. D. (1966). Cystoid Macular Edema and Papilledema Following Cataract Extraction. *JAMA Ophtalmology*.

[8] Ursell P. G., Spalton D. J., Whitcup S. M., Nussenblatt R. B. (1999). Cystoid macular edema after phacoemulsification: Relationship to blood- aqueous barrier damage and visual acuity. *Journal of Cataract & Refractive Surgery*.

[9] Flach AJ. (1998). *The incidence, pathogenesis and treatment of cystoids macular edema following cataract surgery. Trans Am Ophthalmol Soc*.

[10] Rho D. S. (2003). Treatment of acute pseudophakic cystoid macular edema: Diclofenac versus ketorolac. *Journal of Cataract & Refractive Surgery*.

[11] Kim S. J., Equi R., Bressler N. M. (2007). Analysis of macular edema after cataract surgery in patients with diabetes using optical coherence tomography. *Ophthalmology*.

[12] AIOS guidelines to prevent intraocular infection. 2009. Available from http://www.aios.org/guidelinesendoph.pdf.

[13] WHO FAO;. World declaration and plan of action for nutrition.

[14] Dowler J. G. F., Sehmi K. S., Hykin P. G., Hamilton A. M. P. (1999). The natural history of macular edema after cataract surgery in diabetes. *Ophthalmology*.

[15] Lara-Smalling A., Cakiner-Egilmez T. (2014). Diabetes and cataract surgery: preoperative risk factors and positive nursing interventions.. *Insight (American Society of Ophthalmic Registered Nurses)*.

[16] Biro Z., Balla Z., Kovacs B. (2008). Change of foveal and perifoveal thickness measured by OCT after phacoemulsification and IOL implantation. *Eye*.

[17] Şahin Muhammed, Cingü Abdullah Kürşat, Gözüm Nilüfer (2013). Evaluation of Cystoid Macular Edema Using Optical Coherence Tomography and Fundus Autofluorescence after Uncomplicated Phacoemulsification Surgery. *Journal of Ophthalmology*.

[18] Beebe D. C., Shui Y.-B., Siegfried C. J., Holekamp N. M., Bai F. (2014). Preserve the (intraocular) environment: the importance of maintaining normal oxygen gradients in the eye. *Japanese Journal of Ophthalmology*.

[19] Sezgin Akçay B. I., Bozkurt T. K., Guney E., Erdogan G., Ünlü C., Akcali G., Bayramlar H. Quantitative analysis of macular thickness following uneventful and complicated cataract surgery. *Clinical Ophthalmology*.

[20] Chu C. J., Johnston R. L., Buscombe C., Sallam A. B., Mohamed Q., Yang Y. C. (2015). Risk factors and incidence of macular edema after cataract surgery. A database Study of 81984 eyes. *Ophthalmology*.

[21] Liu J., Jones R. E., Zhao J., Zhang J., Zhang F. (2015). Influence of uncomplicated phacoemulsification on central macular thickness in diabetic patients: a meta-analysis. *PLoS ONE*.

[22] Chung J., Kim M. Y., Kim H. S., Yoo J. S., Lee Y. C. (2002). Effect of cataract surgery on the progression of diabetic retinopathy. *Journal of Cataract & Refractive Surgery*.

[23] Henricsson M., Heijl A., Janzon L. (1996). Diabetic retinopathy before and after cataract surgery.. *British Journal of Ophthalmology*.

